# Probing the conformational changes of *in vivo* overexpressed cell cycle regulator 6S ncRNA

**DOI:** 10.3389/fmolb.2023.1219668

**Published:** 2023-07-17

**Authors:** Eleni Makraki, Sophia Miliara, Michalis Pagkalos, Michael Kokkinidis, Efstratios Mylonas, Vasiliki E. Fadouloglou

**Affiliations:** ^1^ Institute of Molecular Biology and Biotechnology, Foundation for Research and Technology—Hellas (IMBB-FORTH), Heraklion, Greece; ^2^ Department of Biology, University of Crete, Heraklion, Greece; ^3^ Department of Molecular Biology and Genetics, Democritus University of Thrace, Komotini, Greece

**Keywords:** non-coding RNA, SAXS, pRNAs, 6S RNA, *in vivo* RNA overexpression

## Abstract

The non-coding 6S RNA is a master regulator of the cell cycle in bacteria which binds to the RNA polymerase-σ^70^ holoenzyme during the stationary phase to inhibit transcription from the primary σ factor. Inhibition is reversed upon outgrowth from the stationary phase by synthesis of small product RNA transcripts (pRNAs). 6S and its complex with a pRNA were structurally characterized using Small Angle X-ray Scattering. The 3D models of 6S and 6S:pRNA complex presented here, demonstrate that the fairly linear and extended structure of 6S undergoes a major conformational change upon binding to pRNA. In particular, 6S:pRNA complex formation is associated with a compaction of the overall 6S size and an expansion of its central domain. Our structural models are consistent with the hypothesis that the resultant particle has a shape and size incompatible with binding to RNA polymerase-σ^70^. Overall, by use of an optimized *in vivo* methodological approach, especially useful for structural studies, our study considerably improves our understanding of the structural basis of 6S regulation by offering a mechanistic glimpse of the 6S transcriptional control.

## Introduction

The field of discovery and characterization of non-coding RNAs (ncRNAs) has been undergoing a rapid expansion in recent years. A prominent member of this class of molecules is 6S RNA, isolated more than 50 years ago (Hindley, 1967) but only relatively recently identified as a key regulator of transcription (Wassarman and Storz, 2000). 6S is found in almost all bacteria (Wehner et al., 2014), occasionally in multiple gene copies (Trotochaud and Wassarman, 2005). *E. coli* 6S is encoded by the *ssrS* gene as a precursor molecule which is processed by RNases to generate the 183 nt mature functional form (Fadouloglou et al., 2015). 6S, while ubiquitously expressed in *E. coli,* is most abundant in late stationary phase (Wassarman, 2007). Recently, it was shown that the 6S levels during the exponential phase of growth of *E. coli* are regulated by the ribonuclease RNase BN (Chen et al., 2016). Due to its high concentration (approximately 10,000 molecules/cell) and affinity for the σ^70^-RNA polymerase (RNAP) holoenzyme (Eσ^70^), 6S RNA inhibits binding of many DNA promoters to RNAP and impedes the transcription from σ^70^-responsive promoters of the majority of genes during stationary phase (Trotochaud and Wassarman, 2004). 6S also influences the levels of the signalling molecule guanosine tetraphosphate which regulates stress responses and growth adaptation (Cavanagh et al., 2010). Moreover, recent evidence from *Rhodobacter sphaeroides* relates 6S gene deletion to a high salt stress phenotype (Elkina et al., 2017). The highly conserved secondary structure of 6S RNA comprises a central domain bulge flanked by two irregular stem structures resembling the structure of an open promoter DNA which enables the formation of stable complexes with RNAP preferentially associated with σ^70^ (Lee et al., 1978; Wassarman and Storz, 2000; Barrick et al., 2005). On the other hand, only weak binding of 6S RNAP is observed to holoenzymes associated with alternative σ factors or the core RNAP (Wassarman and Storz, 2000; Gildehaus et al., 2007; Wassarman, 2007).

**FIGURE 1:**
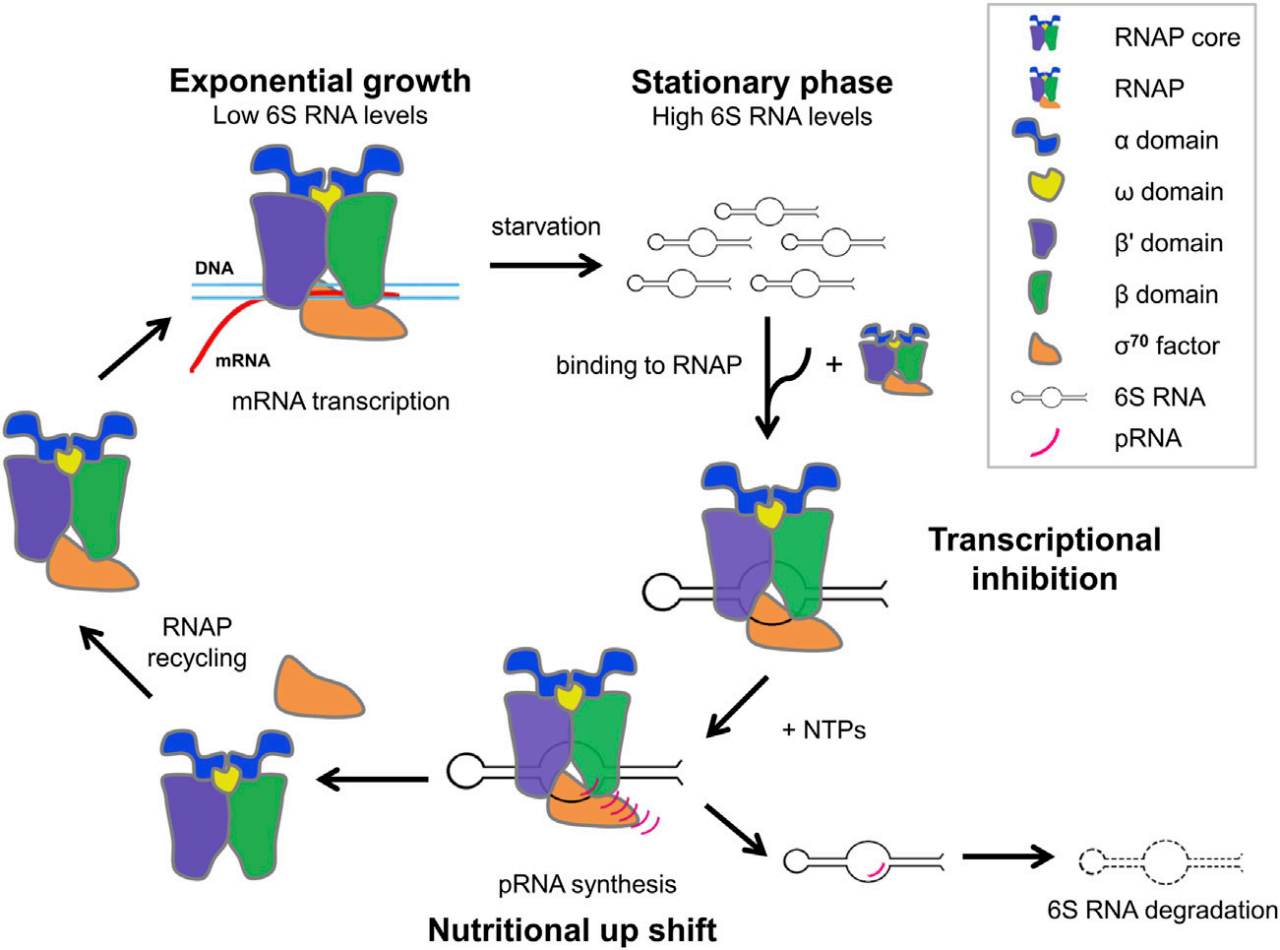
Schematic cycle of growth phase-dependent transcription regulation via 6S RNA during exponential phase, stationary phase and nutritional upshift from stationary phase.

6S RNA has, additionally, the unusual feature to serve as a template for the synthesis of *de novo* transcripts, termed product RNAs (pRNAs) during outgrowth from stationary phase (nutritional upshift) (Wassarman and Saecker, 2006). When pRNA transcripts reach a certain length, pRNAs rearrange the structure of 6S RNA to destabilize the 6S RNA-RNAP complexes resulting in the release of RNAP-bound 6S RNA and restoring regular transcription (Wassarman and Saecker, 2006; Gildehaus et al., 2007; Beckmann et al., 2012; Steuten and Wagner, 2012; Steuten et al., 2014). Mutational studies show that at least three regions of 6S RNA cooperatively interact with the Eσ^70^ to ensure pRNA-dependent release (Oviedo Ovando et al., 2014). 6S RNA remains base-paired to pRNA, released from RNAP as a hybrid (6S RNA: pRNA), thus preventing rebinding of 6S RNA to Εσ^70^ and is subsequently degraded by RNases (Wassarman and Saecker, 2006; Wurm et al., 2010; Beckmann et al., 2012; Cavanagh et al., 2012; Burenina et al., 2014; Chen et al., 2016; Wassarman, 2018) (Figure 1).

Unlike DNA, RNA is much more reactive, often single-stranded which allows it to form intramolecular base-pairing and adopt a variety of three-dimensional structures (e.g., tRNA, rRNA) involved in critical biological processes (Byrne et al., 2010; Pulk and Cate, 2013). These 3D structures are critical for their function, especially in the case of ncRNAs. Due to the limited number of building blocks, the high charge and the tendency of bases to form specific pairs, RNA folding tends to be less complex than protein folding. Nevertheless, the structure of RNA molecules is usually only described in terms of secondary structure, i.e., base pairing, largely ignoring how the three-dimensional arrangement might provide important insights into function. The number of RNA structures in Protein Data Bank (PDB) is only a small fraction (∼3%) of the total deposited structures. This can be partly attributed to the fact that RNA is difficult to work with, unstable and susceptible to degradation by ubiquitous RNases. Moreover, RNA is usually procured from i) endogenous RNA purification which often results in yields insufficient for structural studies or ii) *in vitro* transcription which is especially costly when plentiful supply of high quality RNA material is required. In the case of proteins, this problem is overcome by overexpressing the protein of interest in appropriate hosts.

In this work we explore the three-dimensional conformation of full-length, mature *E. coli* 6S, free and in complex with pRNA with Small Angle X-ray Scattering. To improve the yield of 6S RNA (Fadouloglou et al., 2015), we decided to follow an *in vivo* transcription approach and chromatographic purification protocol, similar to the ones used in protein production and purification (Keel et al., 2009; Ponchon et al., 2009; Nelissen et al., 2012; Baronti et al., 2018; Karlsson et al., 2020). Such approaches have been tested for RNA production and the first reports of successful applications have been published more than three decades ago (Meinnel et al., 1988; Moore et al., 1988; Perona et al., 1988). There have even been optimized applications using structural RNAs as scaffolds to protect sensitive RNAs from RNases (Ponchon and Dardel, 2007; Ponchon et al., 2009; Ponchon and Dardel, 2011). It is surprising, however, that such methodologies are not widely adopted accordingly to their initial success. The technique we propose here is an *in vivo* recombinant overexpression based on cloning into common *E. coli* plasmid vectors, adapted for ncRNAs and especially optimized for 6S production. Purification of the RNA material includes an initial temperature-denaturing step followed by anion-exchange and size-exclusion liquid chromatography. The results were particularly encouraging since we succeeded in producing high yield of high quality 6S RNA. This allowed more comprehensive experiments and significantly improved quality of data. We present here for the first time, the 3D structure of the 6S:pRNA complex using models compatible with SAXS data obtained from Molecular Dynamics calculations. Our models not only are consistent with previous studies on 6S and 6S:pRNA complex (Fadouloglou et al., 2015; Köhler et al., 2015; Ganapathy et al., 2022) but they also account for the flexibility of the nucleic acid particles and provide biophysical evidence for the structural rearrangements that pRNA synthesis induces to 6S and drive its release from RNAP (Chen et al., 2017). Therefore, our work complements and expands on the present knowledge of the molecular mechanisms that govern the gene regulation through non-coding RNAs.

## Results

### Overexpression and purification of full-length *E. coli* 6S

**FIGURE 2:**
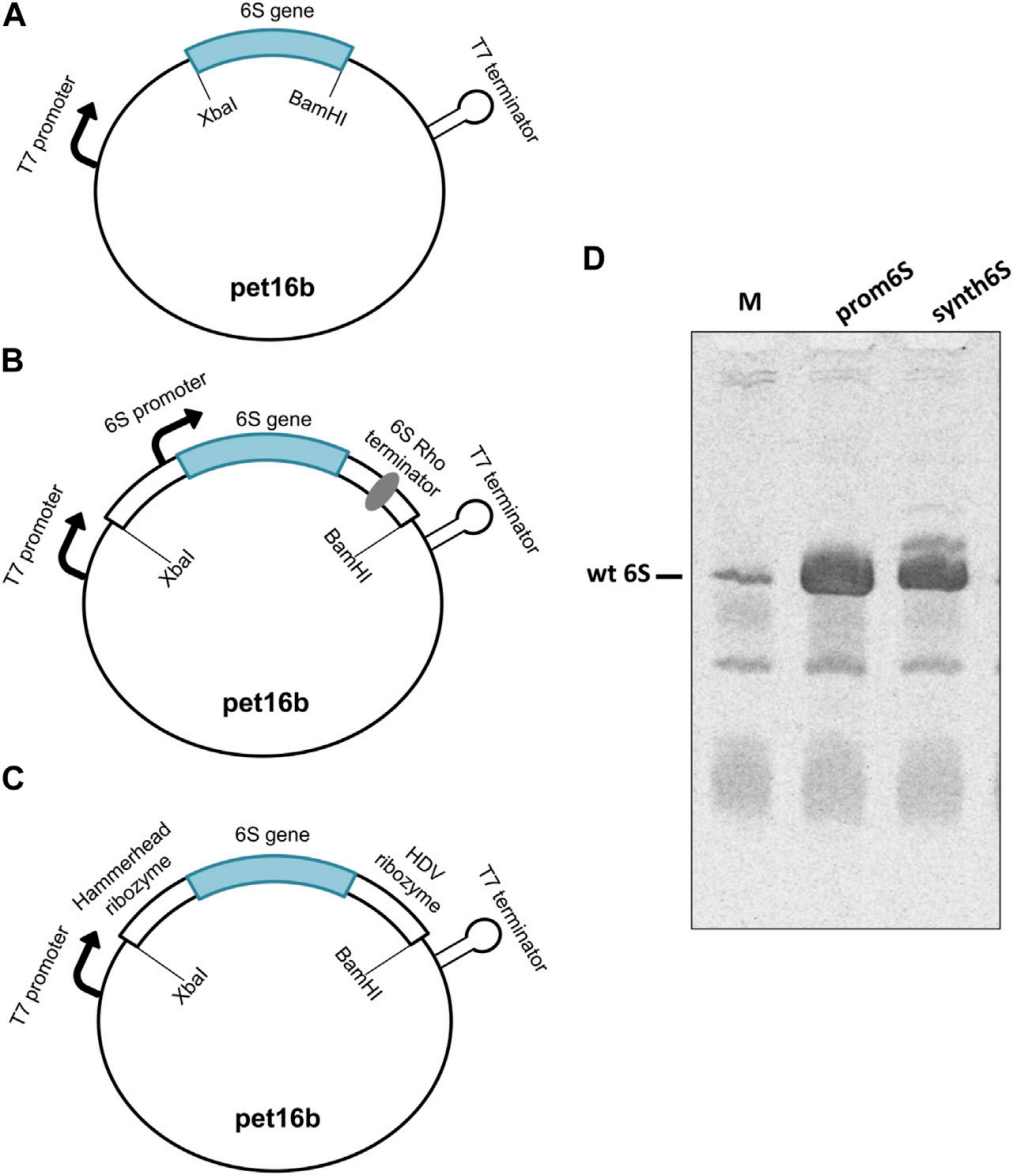
Assessment of constructs designed for 6S RNA production. **(A)** naked6S (mature 6S sequence inserted between two restriction sites in a pet16b vector), **(B)** prom6S (mature 6S sequence with flanking 5′and 3′sequences inserted between two restriction sites in a pet16b vector), **(C)** synth6S (mature 6S sequence with hammerhead and HDV ribozyme sequences in the 5′and 3′sides, respectively, inserted between two restriction sites in a pet16b vector) constructs designed for 6S RNA overexpression in *E. coli*. **(D)** Determination of the overexpression of 6S in BL21 (DE3) competent cells by Urea-PAGE. Lanes: M-molecular weight standard (endogenous BL21 DE3 6S RNA expression, overnight); prom6S- overexpression from prom6S construct, without IPTG, overnight; synth6S- overexpression from synth6S construct, without IPTG, overnight.

6S in *E. coli* is physiologically produced by the *ssrS* gene (Hsu et al., 1985; Chae et al., 2011), controlled by two promoters, the proximal σ^70^-dependent promoter and the distal σ^70^/σ^S^-dependent promoter (Lee et al., 2013). Consequently, two transcripts can be produced, a long precursor of 404 nt and a short precursor molecule of 194 nt. The 5′ end of both precursor transcripts is processed by RNases to produce the 183 nt mature 6S form, hereafter referred to as 6S (Kim and Lee, 2004; Fadouloglou et al., 2015). We cloned the 6S into the pet16b vector and transformed BL21 (DE3) cells, both routinely used for protein overexpression using a T7 polymerase-based system. Three different designs of the vector constructs were examined. The first construct, termed naked6S, contains the mature 6S sequence cloned between two restriction sites. In this design, the transcription initiation is controlled by the T7 promoter and the termination by elements in the vector under the assumption that the RNA is processed by internal RNases to produce the canonical mature 6S form (Figure 2A). The second construct, termed prom6S, contains the mature 6S sequence along with flanking 5′ and 3′ sequences containing both the proximal *ssrS* promoter and most of the precursor 6S sequence on the 5′ end and the rho terminator on the 3′ end. In this manner, the transcription can be controlled by the T7 promoter found upstream of the gene in the vector as well as the proximal *ssrS* promoter while the features that the endogenous *E. coli* RNases recognize to process this chimeric RNA transcript to the mature 6S are retained (Figure 2B). For the last construct, termed synth6S, we followed a drastically different approach independent of *E. coli*-specific processes that can be easily applied to other RNAs. In this construct the mature 6S sequence is flanked by the self-cleaving hammerhead (Prody et al., 1986) and HDV (Kuo et al., 1988) ribozyme sequences at the 5′ and 3′ends respectively. The transcription is controlled by the T7 promoter and the “maturation” of the transcript results from processing by the two ribozymes (Figure 2C).

The naked6S construct did not produce appreciable 6S expression (not shown), possibly reflecting a limited ability of the construct to properly process the transcript to the canonical 6S sequence. On the other hand, both prom6S and synth6S constructs produced high levels of 6S expression higher even than most other RNAs in *E. coli* (Figure 2D; Lanes prom6S and synth6S, respectively), exceeding by far endogenous 6S expression (Figure 2D; Lane M). The synth6S construct showed one more prominent band of a larger size, probably a result of incomplete processing of the self-cleaving ribozymes. For the subsequent experiments, we decided to proceed with prom6S, although the results of the synth6S are very promising and applicable to different types of RNAs.

Bacterial cultures containing the prom6S construct were induced by different IPTG concentrations (0, 0.05, 0.1 and 0.5 mM) at varying durations (1, 2, 4 h and overnight) (Supplementary Figure S1) at 37°C to assess 6S expression. Unexpectedly, the highest 6S expression yield was observed in the overnight cultures with no IPTG addition. The relative intensity of larger RNA bands (e.g., rRNA) was also reduced under these conditions compared to endogenous 6S expression (Figure 2D). Overnight incubation is likely beneficial to 6S expression because more cells enter the static phase increasing its stability. Following these observations, we decided to carry out the 6S RNA expression without adding IPTG at 37°C overnight where the optimum level of 6S RNA was achieved. The 6S RNA purification protocol consisted of two chromatographic steps, i.e., anion exchange (Q-sepharose) and size exclusion chromatography (SEC, Sephacryl S-200) (Nelissen et al., 2012; Edelmann et al., 2014) (Supplementary Figure S2) after heating and refolding of the originally produced RNA material. By this protocol the yield of pure 6S was up to ∼10 mg for a 1 L overnight Terrific Broth culture, orders of magnitude higher than a typical reaction of *in vitro* transcription. In addition, the quality of the product was sufficiently good to allow for the acquisition of excellent quality SAXS data for the structural analysis of 6S. We also examined a longer protocol including hydroxyapatite and arginine-sepharose affinity chromatography. However, the extra steps resulted in a substantially prolonged protocol (and lower yields) without significantly improving the purity of the final RNA.

### SAXS analysis of 6S and the 6S:pRNA complex

**FIGURE 3:**
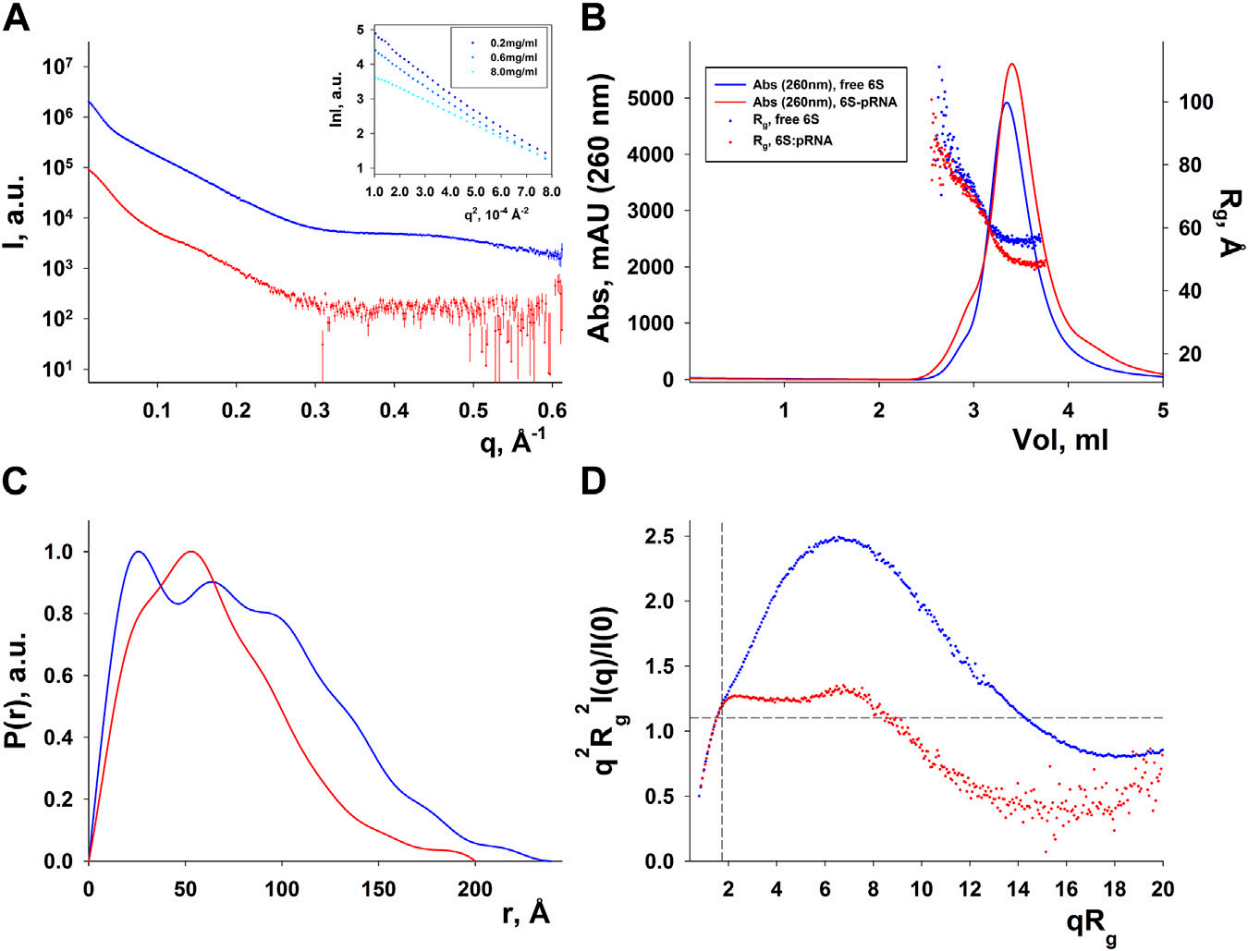
SAXS analysis of free *E. coli* 6S (blue) and in complex with pRNA (red). **(A)** Scattering patterns. The inset shows the Guinier plot of free 6S. **(B)** Chromatograms of the inline SEC-SAXS experiments and scatter plot of the *R*_g_s calculated for the corresponding SAXS frames. **(C)** Distance distribution functions p(r). **(D)** Dimensionless Kratky plots (for a spherical particle, a peak at ∼ q^2^R_g_^2^I(q)/I(0) = 1.1, qR_g_ = 1.732, indicated by dashed lines, is expected).

SAXS data for free 6S and 6S:pRNA were obtained at medium salinity KCl buffers (200 mM), shown in Figure 3A. To prepare the 6S:pRNA complex a chemically synthesized 20 nt RNA (Steuten and Wagner, 2012) was mixed in molar excess (1:3 and 1:7) to the 6S RNA. The samples were also run through an inline size exclusion chromatography system (SEC-SAXS) at the beamline to better assess concentration effects in the SAXS patterns and remove the excess pRNA from the complex (Figure 3B). As expected for strongly negatively charged molecules, there was a decrease of the scattering intensity at higher concentrations due to strong repulsive interactions between the molecules (Figure 3A; inset). The calculated molecular masses were ∼63 kDa and ∼74 kDa, very close to the expected 59 kDa and 66 kDa of the free 6S and the complex, respectively. Conversely, the *R*_g_ were 60 Å and 49 Å for the free 6S and the complex, respectively, indicating a strong compaction of the molecule upon pRNA binding. A shift of the SEC peak to larger elution volumes for the 6S:pRNA complex compared to free 6S indicates a decrease in the hydrodynamic radius of the complex while the homogeneity of the samples is evidenced by the stability of their *R*_g_ throughout their respective peaks (Figure 3B). The distance distribution functions (Figure 3C) further illustrate the compaction occurring upon binding of pRNA as a much smaller *D*_max_ is observed for the complex (240 Å vs. 200 Å). Interestingly, the dimensionless Kratky plot (Durand et al., 2010) of the free 6S corresponds to a typical elongated, very anisometric, “rod-like” particle whereas the dimensionless Kratky plot of the complex corresponds to a less anisometric “disk-like” particle with decreased rigidity, indicating the pRNA synthesis may increase the flexibility of 6S (Figure 3D), possibly as a result of the engorgement of the central domain bulge.

### The elongated 6S RNA collapses in the presence of pRNA

**FIGURE 4:**
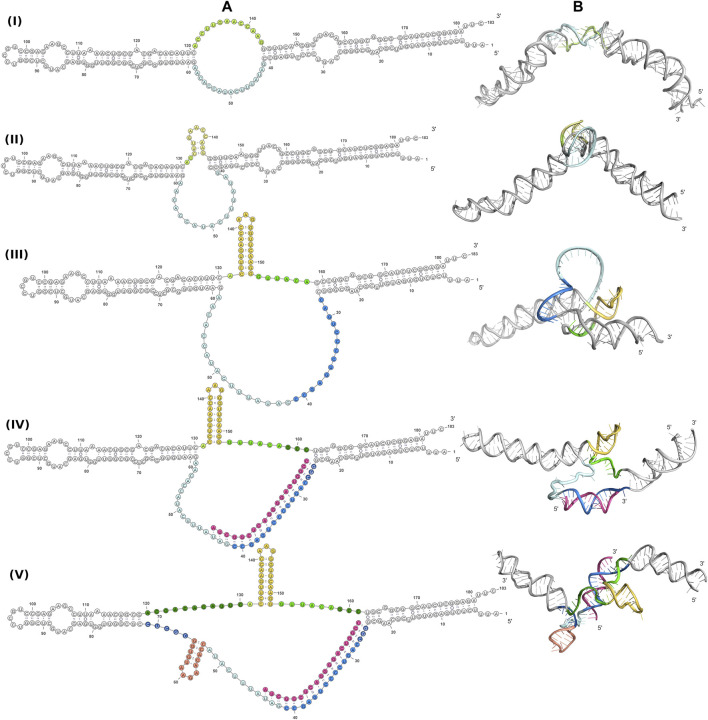
Conformations of free 6S and the 6S:pRNA complex. Secondary structure arrangements **(A)** and the corresponding 3D structures **(B)** of free 6S with no central domain stem loops (fold **I**), with a small central domain 3′stem loop at ∼140 nt (fold **II**), with a large central domain 3′stem loop at ∼140 nt and expanded central domain bulge (fold **III**), and the 6S:pRNA complex without a small 5′stem loop at ∼60 nt (fold **IV**), with a small 5′stem loop (fold **V**). To highlight the differences between the models in the central domain bulge, loops are shown in shades of yellow/orange, features on the 3′of 6S are shown in shades of blue, features on the 3′of 6S are shown in shades of green and pRNA is shown in magenta. Secondary structure illustrations were prepared in VARNA and 3D models in PyMOL (Schrödinger, 2015).

A better understanding of the 6S RNA can be achieved by the analysis of its three-dimensional structure. SAXS, despite being a low resolution method, can discern differences between structures, especially in terms of the overall shape and size of the molecule. Fortunately, RNA has fewer types of building blocks compared to proteins and reasonable three-dimensional models can be built on the basis of secondary structure assumptions. For this purpose, we build 3D models of several secondary structure arrangements of free 6S and the 6S:pRNA complex with the help of RNAComposer (Popenda et al., 2012). The secondary structure arrangements we examined are shown in Figure 4A and declared as folds **I** to **V**. The first free 6S arrangement (fold **I**) contains no stem loops in the central domain bulge (Wassarman, 2007; Chen et al., 2017). The second arrangement (fold **II**) contains a commonly reported (Barrick et al., 2005; Steuten and Wagner, 2012) 3′ small stem loop in the central domain bulge at ∼140 nt (colored in yellow) and the third one (fold **III**) a much larger 3′ stem loop, involving partial unfolding of the closing stem, commonly associated with pRNA synthesis (Chen et al., 2017). For the 6S:pRNA complex, two alternative arrangements were considered differing only in the absence (fold **IV**) or presence of a 5′ stem loop (fold **V**) at ∼60 nt (Panchapakesan and Unrau, 2012; Steuten and Wagner, 2012). The differences between the arrangements may not appear very significant at secondary structure level but they can be very dramatic at tertiary structure level because the small hairpin loops can introduce tensions that completely change the shape and dimensions of the molecule, as shown by the 3D RNAComposer-derived models (Figure 4B).

**FIGURE 5:**
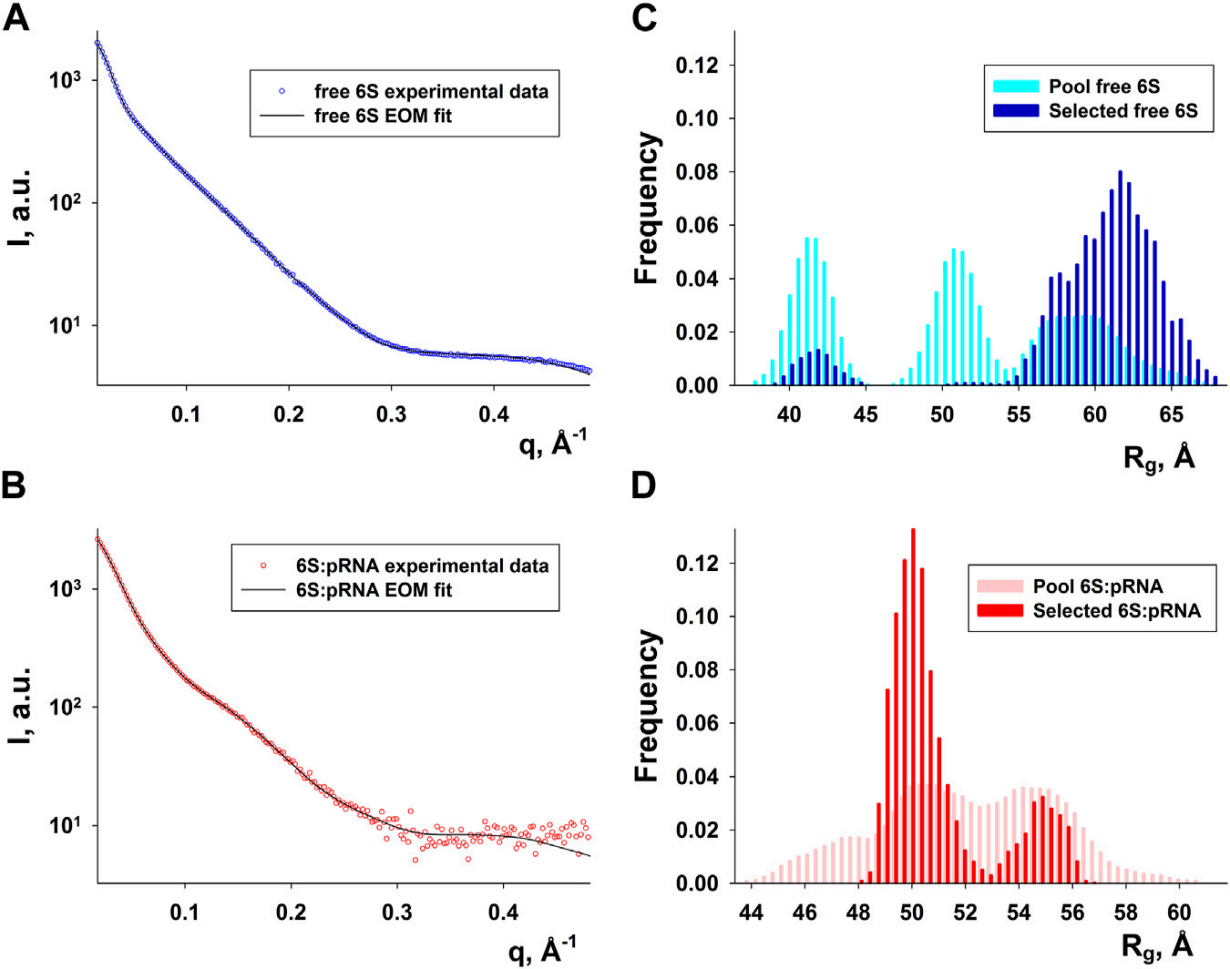
Ensemble fit to the experimental SAXS data of **(A)** free 6S and **(B)** the 6S:pRNA complex. Radius of gyration histograms of the original pool of models vs. the selected ensembles of **(C)** free 6S and **(D)** the 6S:pRNA complex derived from the EOM analysis. Snapshots of the MD simulations starting from the structures shown in Figure 4 comprise the pool of structures from which EOM selects the subset that best describes the SAXS experimental data. The selected ensembles are shown in Supplementary Figures S3, S4 for free 6S and the 6S:pRNA complex, respectively.

**FIGURE 6:**
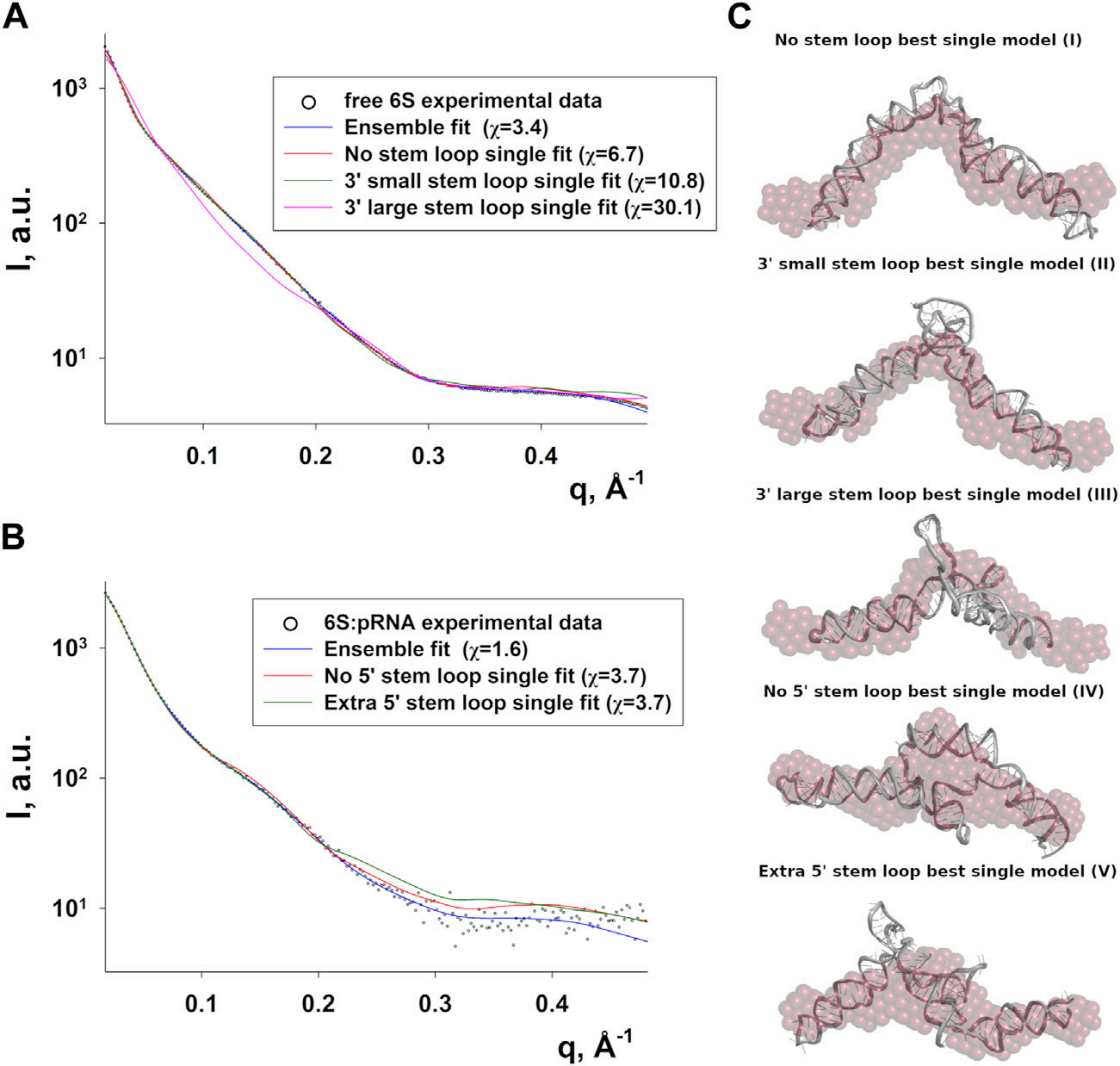
Compatibility of single models with the 6S SAXS data. Fits of the best-fitting single models (MD snapshots) of each fold type to the experimental SAXS data of free 6S **(A)** and 6S:pRNA complex **(B)**, with the improved ensemble (EOM) fits shown for comparison (χ values for each fit shown in the legend). **(C)** The models used for the fits in A and B superimposed to averaged dummy bead models (red) calculated from the SAXS data.

Since RNA is expected to exhibit some structural plasticity, we decided to account for it and expand the conformational space available to the SAXS analysis by running short MD simulations on the RNAComposer-derived models. We used snapshots of the simulation as models, under the constraint of retaining the original secondary structure. These models were used as a pool of structures (each secondary structure arrangement was represented by the same number of structures) from which the EOM program (Bernadó et al., 2007) can select a subset that is consistent with the SAXS data as an ensemble. Interestingly, very good agreement of the ensembles to the experimental SAXS data (Figures 5A, B) was achieved (χ = 3.4 and 1.6 for the free 6S and 6S:pRNA complex, respectively), significantly improved over the best-fitting single models (Figure 6). Representative ensembles are shown in Supplementary Figures S3, S4. Comparison of the *R*_g_ histograms of the selected ensembles vs. the original pool of structures (Figure 5C) illustrates that only the most elongated models (i.e., fold **I**) are compatible with the SAXS data in the case of free 6S, in agreement with our previous work (Fadouloglou et al., 2015) and the 3.8 Å cryo-EM model by Chen et al. (Chen et al., 2017) which clearly shows that 6S adopts fold **I**-like conformation when in complex with Eσ^70^ (without pRNA). Strikingly, no such bias is observed for the 6S:pRNA complex (Figure 5D). To better understand the results, we can look at the composition of the selected ensembles (Table 1; Supplementary Figures S3, S4) with respect to the examined structures (folds **I** to **V**) of Figure 4. Free RNA is predominantly found in a conformation with no stem loops in the central domain bulge, superficially resembling a “fully double helical” structure with some kinks (fold **I**). The hybridization of even a few nucleotides, while looking inconspicuous at the secondary structure level, requires the formation of a small double helix which, in turn, causes a significant compaction of the molecule (fold **II**) incompatible with our experimental data. This effect is even more pronounced in the presence of a larger loop (fold **III**). On the contrary, the best fit to the SAXS data of the complex is achieved when one considers a more equimolar ratio of the two conformations presented in Figure 4B (fold **IV** and fold **V**). Both 6S:pRNA arrangements have a very similar overall shape, despite their differences at the secondary structure level, with extended “double-helical” closing and internal stems and a “swollen” central domain due to the presence of the pRNA. MM-GBSA free energy analysis of the 3D conformations corroborate the above observations (Table 2). While the secondary structure mFold analysis (Table 2) shows no strong preference for the free 6S conformation (with fold **II** having the lowest mFold Δ*G*), the 3D structure free energy of Fold **I** is significantly lower than **II** and **III**, suggesting that it is the most stable free 6S form, in full agreement with the EOM observation. In the case of the 6S:pRNA complex, the free energy difference of the 3D structures of the two conformations is smaller (while the mFold difference is larger than in the case of free 6S), with fold **IV** being preferable to fold **V**, also similar to the EOM analysis. Binding free energy analysis of pRNA to 6S (Table 2) shows that pRNA binding is favorable for both fold **IV** and **V**.

**TABLE 1:** Ensemble population analysis. Each population corresponds to the respective secondary structures shown in Figure 4A. Typical ensembles are shown in Supplementary Figures S3, S4.

Free 6S	
No stem loop (fold **I**)	91% ± 2%
3′small stem loop (fold **II**)	2% ± 2%
3′large stem loop (fold **III**)	7% ± 3%
6S:pRNA complex	
No 5′stem loop (fold **IV**)	70% ± 3%
Extra 5′stem loop (fold **V**)	30% ± 3%

**TABLE 2:** Free energies calculated for the secondary (mFOLD) and tertiary (MM-GBSA) structures of free 6S/6S:pRNA complex conformations. The binding free energy of the pRNA to the complexes (MM-GBSA) is also shown.

	Conformation	mFOLD Δ*G* (kcal/mol)	MM-GBSA *G* (kcal/mol)	pRNA binding Δ*G* (kcal/mol)
free 6S	No stem loop (fold **I**)	−77.10	−37527.48	-
	3′small stem loop (fold **II**)	−78.53	−37350.35	-
	3′large stem loop (fold **III**)	−74.97	−37402.33	-
6S:pRNA complex	No 5′stem loop (fold **IV**)	−117.11	−41710.22	−190.02
	Extra 5′stem loop (fold **V**)	−112.38	−41675.96	−178.11

**FIGURE 7:**
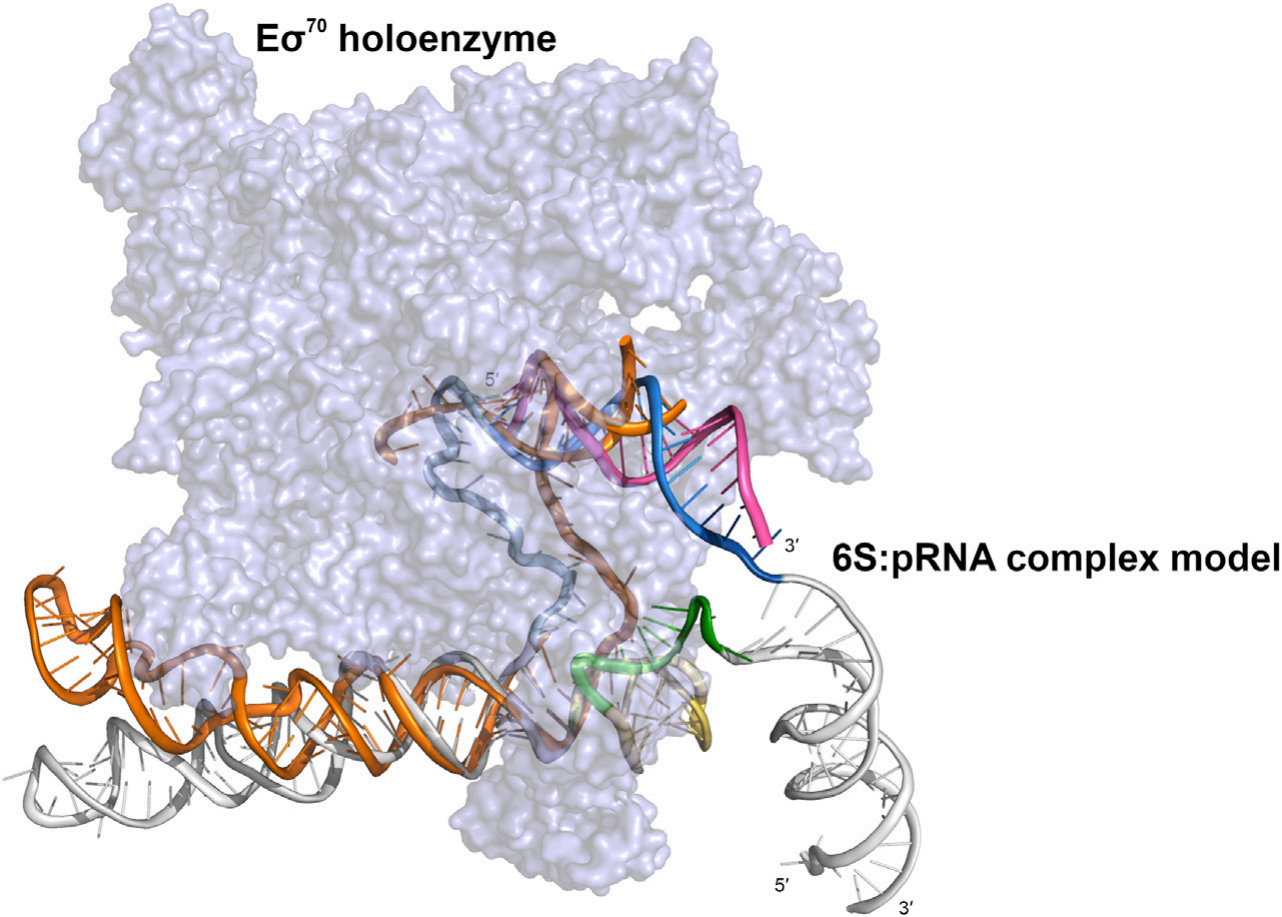
Cartoon representation of a 6S:pRNA complex model fitted inside the cryo-electron microscopy structure of the Eσ^70^:6S complex (PDB ID 5VT0). Eσ^70^ shown in purple surface representation and 6S in orange cartoon representation. The 6S:pRNA model fitted into the Eσ^70^ holoenzyme is derived from a 6S:pRNA complex model that contains no extra loop at ∼60 nt (fold **IV**), using the same color scheme. The figure was created in PyMOL (Schrödinger, 2015).

The free, flexible, fold **I**-like 6S can accommodate the structural changes required for the change from the solution form to the Eσ^70^–bound open promoter-like form (Barrick et al., 2005; Chen et al., 2017) while this is hindered by the “swollen” central domain of the 6S:pRNA complex and the reduced structural plasticity, imposed by the presence of the pRNA. In Figure 7, we superimpose on the 6S:Eσ^70^ experimental model (Chen et al., 2017) a fold **IV** model of the 6S:pRNA complex in order to compare the two predominant states of 6S (Table 1, fold **I** and fold **IV**) for their fit into the Eσ^70^. The presence of the 3′ stem loop at ∼140 nt (Figure 7, colored in yellow) very likely makes the 6S shape incompatible with binding to Eσ^70^ and additionally, withdraws a significant number of nucleotides from interactions with the protein by engaging them to nucleotide pairing interactions. In the 6S:Eσ^70^ experimental model (Chen et al., 2017) the nucleotides U134-G143 are single stranded and in direct interaction with the protein. These same nucleotides are the ones that form the 3′stem loop. Thus, the 6S:pRNA complex can hardly fit into the Eσ^70^ cavity (Figure 7) when 6S adopts fold **IV** (and impossible when adopts fold **V**) and even then it would first require the disengagement of σ^70^ from RNA polymerase. This renders the re-integration of the complex back to the Eσ^70^ holoenzyme very unlikely, rescuing it after pRNA transcription and allowing it to resume transcription of genes.

## Discussion

The paradigm shift of the central dogma of the role of RNA in the cell as messenger, transfer or ribosomal RNA has it now implicated in a multitude of processes in regulatory or enzymatic roles (Scott, 2007; Storz et al., 2011; Breaker, 2018). 6S is one such molecule, a master regulator of transcription being highly conserved in most bacterial families (Wehner et al., 2014). We often tend to think of RNA as a two-dimensional molecule and consequently few attempts have been made for a more comprehensive analysis of 6S with structural methods. These include the characterization of mature and precursor *E. coli* 6S with biophysical methods (Fadouloglou et al., 2015), the Nuclear Magnetic Resonance analysis of *Aquiflex aeolicus* 6S (Köhler et al., 2015), the cryo-electron microscopy study of the complex of *E. coli* Eσ^70^ with 6S (Chen et al., 2017) and the recent work on the *Bacillus subtilis* 6S with Atomic Force Microscopy (AFM) (Ganapathy et al., 2022).

Although methods for *in vivo* RNA overexpression (Meinnel et al., 1988; Moore et al., 1988; Perona et al., 1988) and purification with chromatographic and affinity methods were presented in the past (Martins et al., 2010; Panchapakesan et al., 2017), they are not widely employed. Conversely, RNA production is routinely performed with *in vitro* transcription, an expensive and inefficient process rendering it significantly more costly and laborious than protein-based products for the same applications (even if one considers in-house overexpressed T7/SP6 polymerase). Since the supply of pure as well as chemically and structurally homogeneous RNA samples in mg amounts is a prerequisite for structural studies, we decided first to develop an optimised methodology for *in vivo* overexpression of 6S and purification under mostly native conditions. Various 6S constructs were designed and tested for best results. After refinement of growth conditions, we achieved excellent overexpression of 6S. The product has the same electrophoretic behavior as the endogenous material and produces a strong sharp band with Urea-PAGE. Our protocol is superior to commonly applied *in vitro* transcription methods used for RNA production at the following points: i) it readily produces exceptionally large amounts of material; ii) the cost per milligram of pure RNA material is substantially low, at least two orders of magnitude cheaper than *in vitro* transcription kits, comparable with the cost per milligram of pure protein material. iii) The produced RNA material is highly pure and homogeneous as can be judged by Urea-PAGE and size-exclusion chromatography. Accordingly, we have illustrated that *in vivo* overexpression of RNA is an approach that merits further exploration, e.g., by using stable structured RNAs such as tRNAs (Ponchon and Dardel, 2007), 5S (Zhang et al., 2009) or even 6S as scaffolds to “protect” other RNAs by nucleases during the conventional fermentation of *E. coli*. We expect that our results will encourage more widespread adoption and exploration of *in vivo* RNA overexpression methods especially now that RNA has come to the spotlight as a new biotechnological product for several health-related applications such as the mRNA vaccines for SARS-CoV2 (Polack et al., 2020; Baden et al., 2021) and the auspicious results for mRNA cancer vaccines (Miao et al., 2021). Certainly, there are still hurdles to be overcome. Larger mRNA molecules will likely be much more brittle than the smaller and structured 6S RNA and modified nucleotides such as those contained in the SARS-CoV2 mRNA vaccines (Morais et al., 2021; Nance and Meier, 2021; Rosa et al., 2021) may be difficult to incorporate in an *in vivo* protocol.

The high yield and quality 6S material produced by our improved *in vivo* overexpression protocol allowed for the acquisition of high quality SAXS data and the characterization of the *E. coli* 6S and its structural transition after pRNA binding. Our structural analysis confirms a highly elongated shape for the free *E. coli* 6S RNA (Fadouloglou et al., 2015) and suggests that this shape is probably conducive to Eσ^70^ binding since it allows easier access to the RNAP cavity after which it can adopt an open promoter complex-like structure (Chen et al., 2017). The presence of pRNA molecules completely changes the conformation of the central domain, swelling it and making it more unstable and, ultimately, most likely incompatible with the RNAP cavity. These observations are also in agreement with an AFM analysis in *Bacillus subtilis* (Ganapathy et al., 2022), hinting at a universal mechanism of 6S conformational change and release. Moreover, the conformational shift of the 6S:pRNA complex occurred even with a modest molar excess of pRNA (1:3) and “survived” the passage through a size exclusion chromatography column inline with SAXS to separate from excess pRNA. This suggests that the binding is quite strong and energetically favorable which is further supported by the MM-GBSA binding free energy analysis (Table 2).

Our current hypothesis, combining all the available data, is that the Eσ^70^:6S:pRNA triple complex is biophysically unstable and the 6S:pRNA interactions are thermodynamically preferred over the 6S: Eσ^70^ ones. Furthermore, the 6S:pRNA 3D conformation is probably incompatible with Eσ^70^ binding, especially due to the 3′ stem loop formation at ∼140 nt. Although the 6S:Eσ^70^ complex is stable, the progressive increase of the pRNA length dramatically changes both the 6S RNA shape and, consequently, the 6S RNA affinity for Eσ^70^. In particular, gradual synthesis of pRNA causes the gradual unfolding of the double stranded 6S (fold **I**-like). The one strand serves as a pRNA template while the other strand is released and forms a 3′ stem loop at ∼140 nt. A more compact 6S particle is gradually formed and at the same time nucleotides U134-G143, which were initially single-stranded and involved in interactions with Eσ^70^, are engaged in the stem’s double helix formation, consequently unable to participate in interactions with the protein. Therefore, favorable 6S:Eσ^70^ interactions are lost and the overall fit of 6S inside Eσ^70^ worsens. Once pRNA reaches a certain length the above described phenomena reach a breaking point. The significant conformational changes in conjunction with the displacement of 6S due to transcription and the disruption of several 6S:Eσ^70^ interactions lead to the release of the 6S:pRNA particle from Eσ^70^. On the other hand, 6S is unlikely to dissociate from pRNA until its degradation, explaining why, even though 6S is constitutively expressed, it has no effect during the exponential growth phase. In other words, once pRNA of a certain length (Beckmann et al., 2012) is synthesized, 6S remains permanently bound to it, rendering it unable to rebind to RNA polymerase, until its degradation (Wassarman and Saecker, 2006; Gildehaus et al., 2007; Wurm et al., 2010; Chen et al., 2016).

In summary, we provide evidence by Small Angle X-ray Scattering analysis that complex formation between *E. coli* 6S RNA and pRNA is associated with a compaction of the overall 6S size and an expansion of its central domain bulge. Similar observations for the *Bacillus subtilis* 6S-1 RNA (Ganapathy et al., 2022) indicate that structural rearrangements induced by pRNA synthesis to 6S RNA may be a common property of non-coding 6S RNA cell cycle regulators and suggest a simple, elegant and universal mechanism of transcription control based on the abundance of nucleotides. The importance of this structural switch could be further supplemented by future experimental work on the stability and dynamics of the Eσ^70^:6S complexation, in the presence and absence of pRNA.

## Materials and methods

### Vector construction

Three different constructs were designed for overexpression in *E. coli* and cloning in the pet16b vector (Figures 2A–C) between the XbaI and BamHI restriction sites. The first construct, naked6S, only contains the mature 6S sequence between the two restriction sites. The second construct, prom6S, contains the mature 6S sequence as well as 5′ and 3′ flanking sequences, including the proximal promoter of the 6S gene. The third construct, synth6S, was synthesized (integrated DNA Technologies) and the mature 6S sequence is incorporated between two self-cleaving ribozymes; 5′ hammerhead (Prody et al., 1986) and 3′ HDV (Kuo et al., 1988) (shown in italics below). All sequences were verified by sequencing. Restriction sites are shown in bold.

Mature 6SRNA sequence:

5′-AUU​UCU​CUG​AGA​UGU​UCG​CAA​GCG​GGC​CAG​UCC​CCU​GAG​CCG​AUA​UUU​CAU​ACC​ACA​AGA​AUG​UGG​CGC​UCC​GCG​GUU​GGU​GAG​CAU​GCU​CGG​UCC​GUC​CGA​GAA​GCC​UUA​AAA​CUG​CGA​CGA​CAC​AUU​CAC​CUU​GAA​CCA​AGG​GUU​CAA​GGG​UUA​CAG​CCU​GCG​GCG​GCA​UCU​CGG​AGA​UUC-3′

Naked6S primers:

P1: 5′-GTG​GGC**TCT​AGA**ATT​TCT​CTG​AGA​TGT​TCG​CAA​GC-3′

P2: 5′-GTT​GAT**GGA​TCC**GAA​TCT​CCG​AGA​TGC​CGC​C-3′

Prom6S primers:

P1: 5′-GTG​GGC**TCT​AGA​C**TAC​GCG​GCA​AGT​ATG​GAA​C-3′

P2: 5′-GTT​GAT**GGA​TCC**GAG​AGG​AAT​ACA​GCG​ACC​GT-3′

Synthetic Gene with flanking HammerHead and HDV ribozymes:

5′-**TCT​AGA** *GGG​AGA​GAG​AAA​TCT​GAT​GAG​TCC​GTG​AGG​ACG​AAA​CGG​TAC​CCG​GTA​CCG​TC*ATT​TCT​CTG​AGA​TGT​TCG​CAA​GCG​GGC​CAG​TCC​CCT​GAG​CCG​ATA​TTT​CAT​ACC​ACA​AGA​ATG​TGG​CGC​TCC​GCG​GTT​GGT​GAG​CAT​GCT​CGG​TCC​GTC​CGA​GAA​GCC​TTA​AAA​CTG​CGA​CGA​CAC​ATT​CAC​CTT​GAA​CCA​AGG​GTT​CAA​GGG​TTA​CAG​CCT​GCG​GCG​GCA​TCT​CGG​AGA​TTC*GGG​TCG​GCA​TGG​CAT​CTC​CAC​CTC​CTC​GCG​GTC​CGA​CCT​GGG​CTA​CTT​CGG​TAG​GCT​AAG​GGA​GAA​G* **GGA​TCC-**3′

### *In vivo* RNA production

The recombinant plasmid was transformed into *E. coli* BL21 (DE3) competent cells for RNA expression. A single colony was cultivated in 20 mL LB medium containing 50 μg/mL ampicillin (LB amp^50^) at 37°C, 250 rpm and used to inoculate 2 L of Terrific Broth Medium (TB) containing 50 μg/mL ampicillin (TB amp^50^). It was subsequently incubated overnight at 37°C, 250 rpm. The cells were pelleted and washed with 20 mM Tris-HCl pH 7, 200 mM NaCl. The RNA was isolated by acid-guanidinium thiocyanate-phenol-chloroform extraction in an analogous manner to that previously described by Chomczynski (Chomczynski and Sacchi, 2006). The RNA was analyzed by electrophoresis on an analytical denaturing 7.2% urea polyacrylamide gel (Urea-PAGE) in 1X TBE containing 8M urea (Figure 2D).

### RNA purification

RNA pellets from 2 L of culture were diluted to 300 mL of a buffer containing 50 mM K_2_HPO_4_/KH_2_PO_4_ pH 7.0 and 0.1 mM EDTA, heated to 95°C for 5 min and subsequently placed on ice for 20 min to refold and centrifuged to precipitate protein remains. RNAs were purified using an Akta purifier system (Amersham), Q Sepharose anion exchange column and a Sephacryl S-200 column (GE Healthcare). The Q Sepharose column was pre-equilibrated with buffer A (20 mM Tris HCl pH 8.0, 400 mM NaCl, 100 mM KCl, 0.1 mM EDTA). After loading the sample onto the column 400 mL of buffer A was used to wash the column and subsequently a 800 mL gradient of buffer B (20 mM Tris HCl pH 8.0, 620 mM NaCl, 100 mM KCl, 0.1 mM EDTA) up to 100% (620 mM NaCl) was performed while collecting 8.5 mL fractions. The system was cleaned with 200 mL of 100% buffer B to wash off remaining uncleaved RNA and DNA remains. Absorbance was monitored continuously at 260 nm. The fractions were analyzed by 7.2% Urea-PAGE in 1X TBE containing 8 M urea. Fractions with the 6S RNA were merged, heated to 95°C for 5 min and subsequently placed on ice for 20 min to refold. After centrifugation, the sample was loaded onto a Sephacryl S-200 column (GE Healthcare), and 6S RNA eluted with a buffer containing 20 mM Tris HCl pH 8.0, 200 mM KCl and 5 mM MgCl_2_. The purified 6S RNA was concentrated by ultrafiltration with 10 kDa cut-off filter and analyzed by 7.2% Urea-PAGE in 1X TBE containing 8 M urea. RNA quantification was performed as described before (Chomczynski and Sacchi, 2006).

### 6S:pRNA complex formation

The pRNA in our experiments is the 20 nt synthetic sequence 5′-AUC​GGC​UCA​GGG​GAC​UGG​CC-3′ (Steuten and Wagner, 2012). For the complex formation, purified 6S RNA solution (in 20 mM Tris HCl pH 8.0, 200 mM KCl and 5 mM MgCl_2_ buffer) was mixed with molar excess of pRNA in ratios 1:3 and 1:7 to a final volume of 75 μL. The mixture was heated at 94°C for 4 min and subsequently placed at 37°C for 1.5 h.

### Small angle X-ray scattering measurements and primary data analysis

Preliminary SAXS data of 6S were collected at the EMBL Hamburg P12 undulator beamline of the Petra III storage ring in DESY (Hamburg, Germany) using a Pilatus 2M (DECTRIS) photon counting pixel detector (Blanchet et al., 2015). The measurements were performed at 10°C using the automated sample changer. The sample-to-detector distance was 3.1 m, covering a range of momentum transfer 0.02 < s < 4.8 nm^−1^ (s = 4π sinθ/λ, where 2θ is the scattering angle, and λ = 1.24 Å is the X-ray wavelength). Primary data reduction, radial averaging, averaging and subtraction were performed on-site with the beamline software (SASFLOW, v. 3.0, Hamburg, Germany). The majority of SAXS data of 6S as well as the 6S:pRNA complex were collected at the SWING Beamline of Synchrotron SOLEIL (Gif-sur-Yvette, France) with an Aviex charge-coupled device detector (David and Pérez, 2009). The measurements were performed at 15°C for several different concentrations of 6S (up to 8 mg/mL) using the automatic sample changer. Both free 6S and 6S:pRNA complex samples were also run through an Agilent HPLC system with a gel filtration column to assess the behavior of the samples at lower effective concentrations and for the 6S:pRNA complex to remove unbound pRNA. The sample-to-detector distance was 3.1 m, covering a range of momentum transfer 0.007 < q < 0.614 Å^−1^ (*q* = 4*π* sin*θ*/*λ*, where 2*θ* is the scattering angle, and λ = 1.033 Å is the X-ray wavelength). Using the Foxtrot software, the data were averaged radially and converted to absolute units, analyzed for radiation damage, averaged and subtracted. Subsequent analysis was performed with the ATSAS program suite (Petoukhov et al., 2012). PRIMUS (Konarev et al., 2003) was used to merge data from different concentrations, and for the calculation of the radius of gyration *R*_g_ and the forward scattering intensity *I*(0) (proportional to the number of electrons of the particle) from the slope of Guinier plot (ln*I*(*q*) versus *q* ^2^) (Guinier, 1939). GNOM (Svergun, 1993) was used to calculate the pair distance distribution function *p*(*r*) and to estimate the maximum particle dimension (*D*_max_). The molecular mass (MM) of the solute was estimated from the SAXS data from the *I(0)*. Twenty dummy beads models of the SAXS data were created for both free 6S and 6S:pRNA complex with DAMMIF (Franke and Svergun, 2009) and averaged with DAMAVER (Volkov and Svergun, 2003).

### 3D structural modeling of SAXS data

A few alternative secondary structure arrangements (illustrations produced with the VARNA program) (Darty et al., 2009) of free 6S and 6S:pRNA complex were produced and the corresponding free energies (Δ*G*) were calculated with mFold (Zuker, 2003). These structures were used as input for the online 3D structure prediction program RNAComposer (Popenda et al., 2012). To better assess the conformational space explored by the molecules, short (100 ns) all-atom Molecular Dynamics simulations were performed with the AMBER14 program suite (Case et al., 2005) with the parameters of the ff14SB force field (Maier et al., 2015). All simulations were performed in 2 fs steps at 300K and constant pressure of 1 atm in TIP3P water boxes with periodic boundary conditions after an initial minimization and stepwise heating in constant volume. Snapshots of the simulations were taken at regular intervals and the SAXS patterns were calculated with CRYSOL (Svergun et al., 1995). The Ensemble Optimization Method (EOM) program (Bernadó et al., 2007) was used to select the subset of models with the best fit to the experimental SAXS data. *R*_g_ values of the models were also calculated and used to create histograms to compare, in a general manner, the population of the pool of original models (i.e., all the models) vs. the subset of the selected ensembles. MM-GBSA free energies (G) and binding free energies of pRNA to the 6S:pRNA complex (Δ*G*) were calculated with the MMPBSA module (Miller et al., 2012) of AMBER14 from 20 snapshots taken from the last 20ns of each simulation.

## Data Availability

The datasets presented in this study can be found in online repositories. The names of the repository/repositories and accession number(s) can be found below: https://www.sasbdb.org/data/SASDQR4, https://www.sasbdb.org/data/SASDQS4.
