# Supplementary figures and images for: Probing the conformational changes of in vivo overexpressed cell cycle regulator 6S ncRNA
Source: Front Mol Biosci. 2023 Jul 17;10:1219668. doi: 10.3389/fmolb.2023.1219668 (PMC10406553; doi:10.3389/fmolb.2023.1219668)

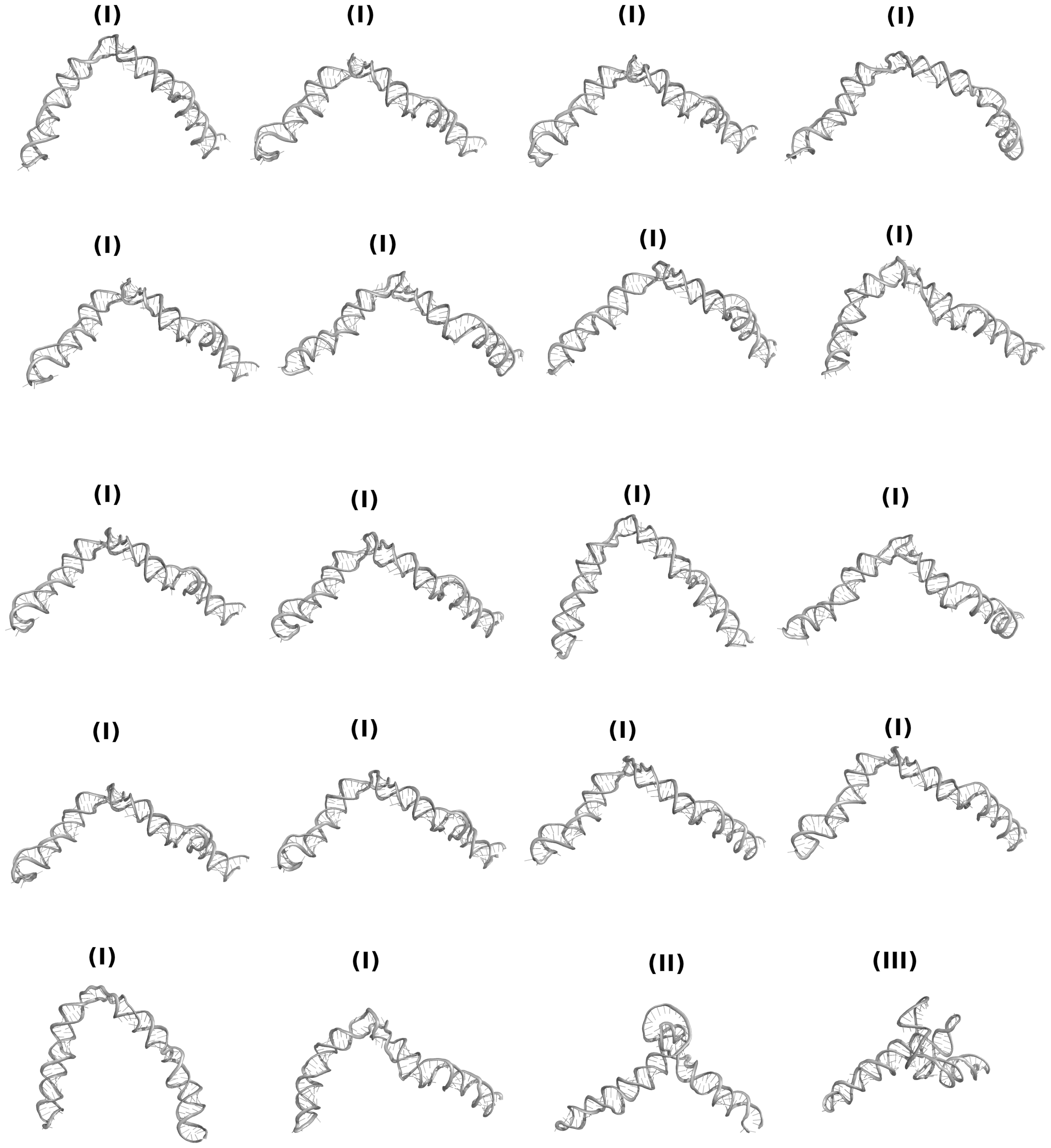

Supplement: Supplementary file 1 [file Image3.TIF]

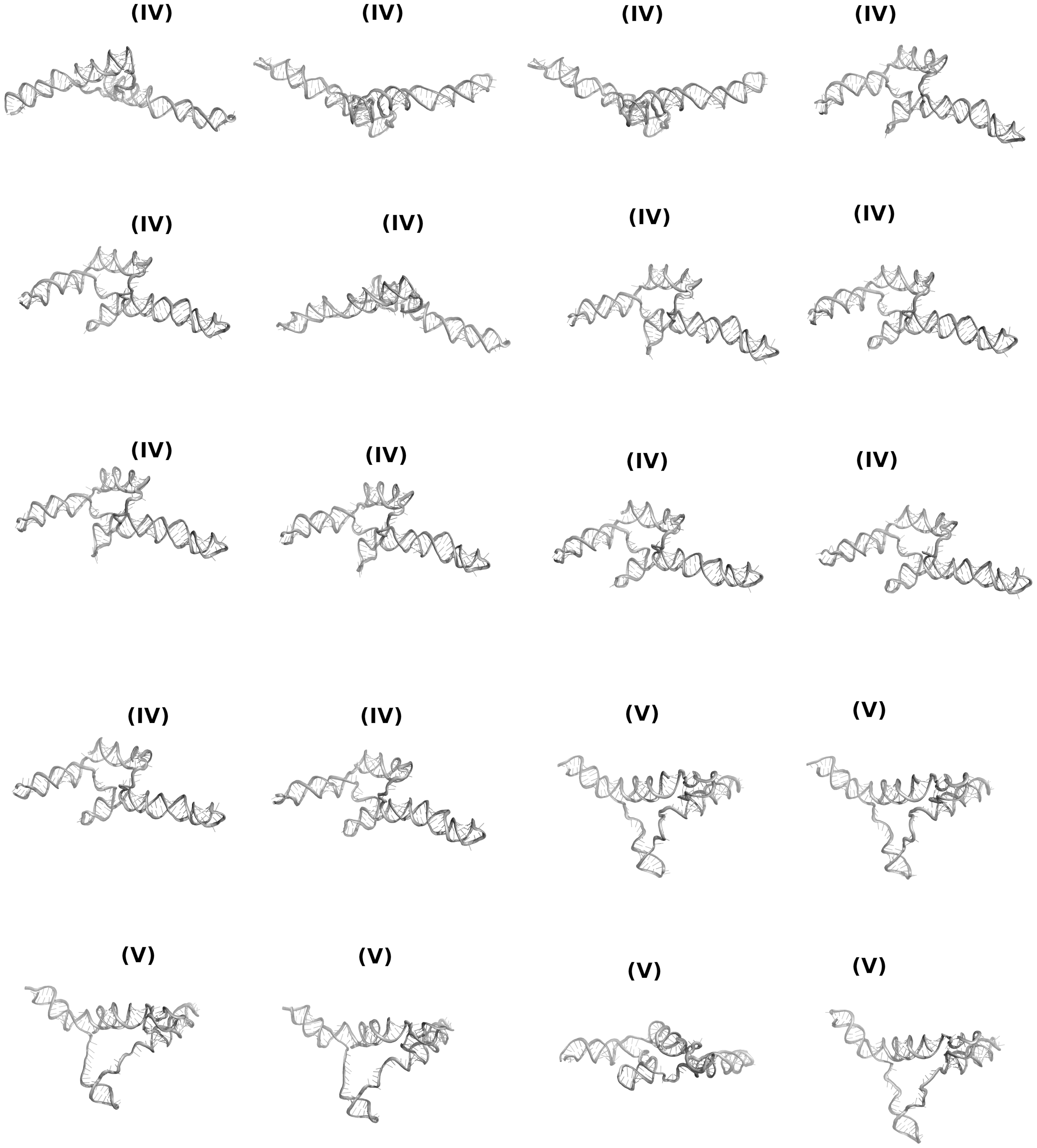

Supplement: Supplementary file 2 [file Image4.TIF]

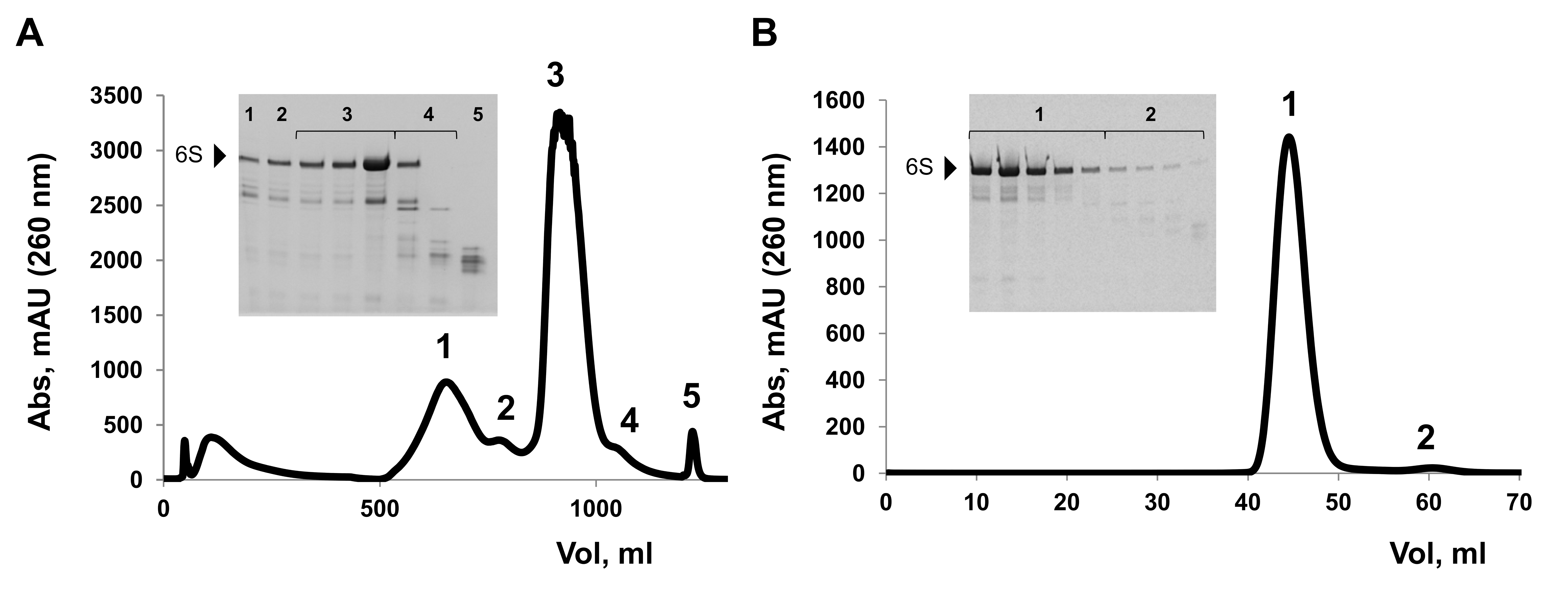

Supplement: Supplementary file 3 [file Image2.TIF]

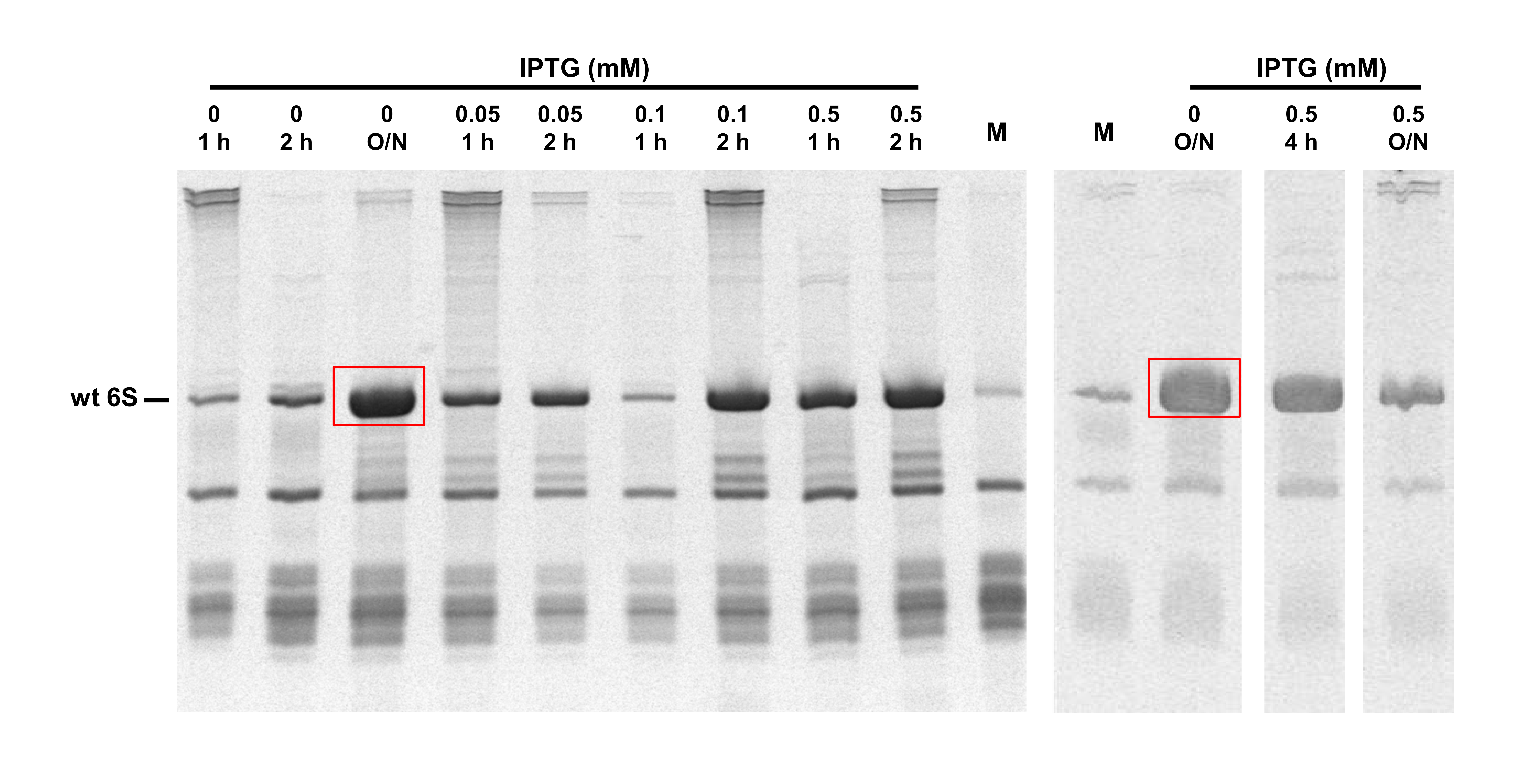

Supplement: Supplementary file 4 [file Image1.TIF]
