# Supplementary material for: Probing the conformational changes of in vivo overexpressed cell cycle regulator 6S ncRNA
Source: Front Mol Biosci. 2023 Jul 17;10:1219668. doi: 10.3389/fmolb.2023.1219668 (PMC10406553; doi:10.3389/fmolb.2023.1219668)
Supplement: Supplementary file 5 [file DataSheet1.pdf]

Supplementary Material

Probing the conformational changes of *in vivo* overexpressed cell cycle regulator 6S ncRNA

Eleni Makraki[1], Sophia Miliara[1], Michalis Pagkalos[1, 2], Michael Kokkinidis[1, 2], Efstratios Mylonas[1,*], Vasiliki E. Fadouloglou[1,3,*]

*To whom correspondence should be addressed. Tel: +30 2551030640; Email: fadoulog@mbg.duth.gr. Correspondence may also be addressed to tel: +30 2810394429; Email: stratos_mylonas@imbb.forth.gr.

Supplementary Figure S1. Optimization of prom6S construct expression in *E.coli* BL21 (DE3) cells.

Supplementary Figure S2. 6S RNA purification.

Supplementary Figure S3. A representative ensemble of free 6S models compatible with the corresponding SAXS data.

Supplementary Figure S4. A representative ensemble of 6S:pRNA models compatible with the corresponding SAXS data.

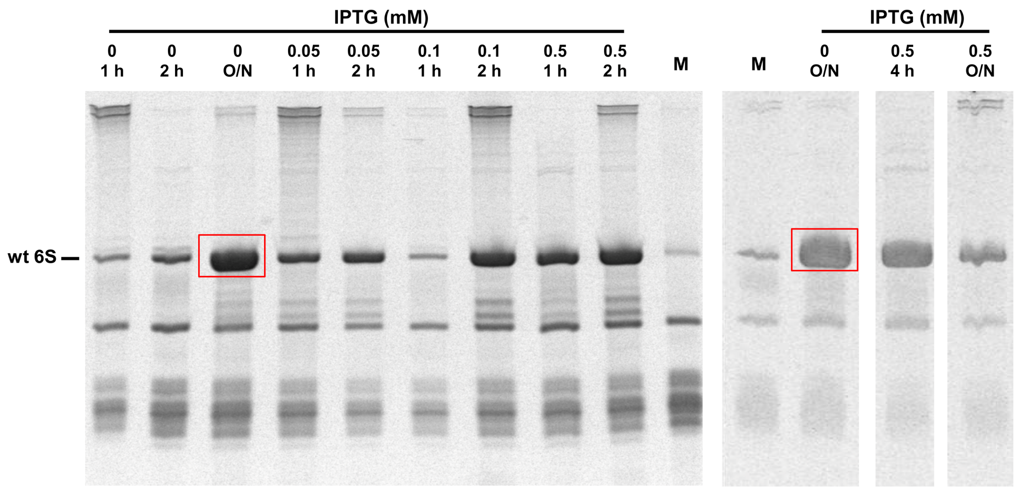

Supplementary Figure S1. Optimization of prom6S construct expression in *E.coli* BL21 (DE3) cells. Effect of induction on expression of 6S at different IPTG concentrations (0 to 0.5 mM) and time points (1 h to overnight) at 37°C was determined by Urea-PAGE. The concentrations of IPTG and time points are shown on the top of the lanes. Lane: M-molecular weight standard (endogenous BL21 (DE3) 6S expression, overnight). The optimal expression condition is indicated with a red frame.

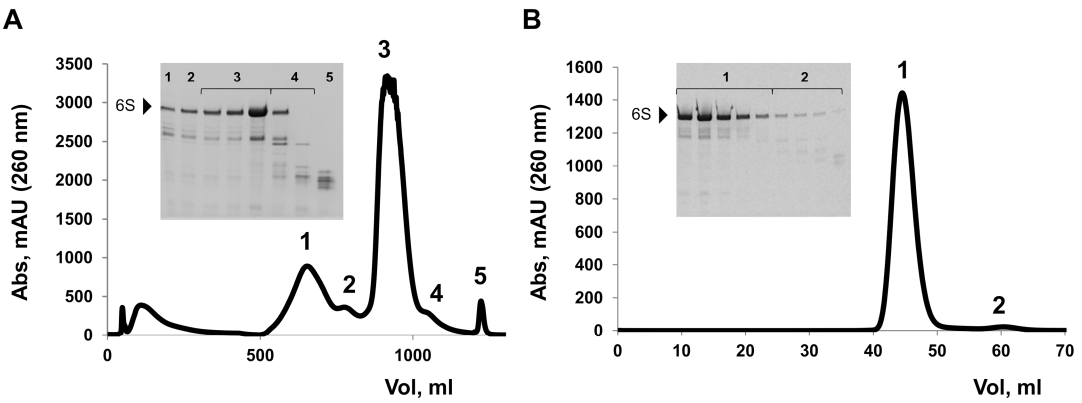

Supplementary Figure S2. 6S RNA purification. A) Chromatogram of the anion exchange chromatography. Fractions of peak 3 were pooled and loaded onto the next column. B) Chromatogram of the size exclusion chromatography. The insets show the electrophoretic Urea-PAGE profiles of the chromatographic peaks as indicated by the corresponding numbers. The arrows indicate the 6S band.

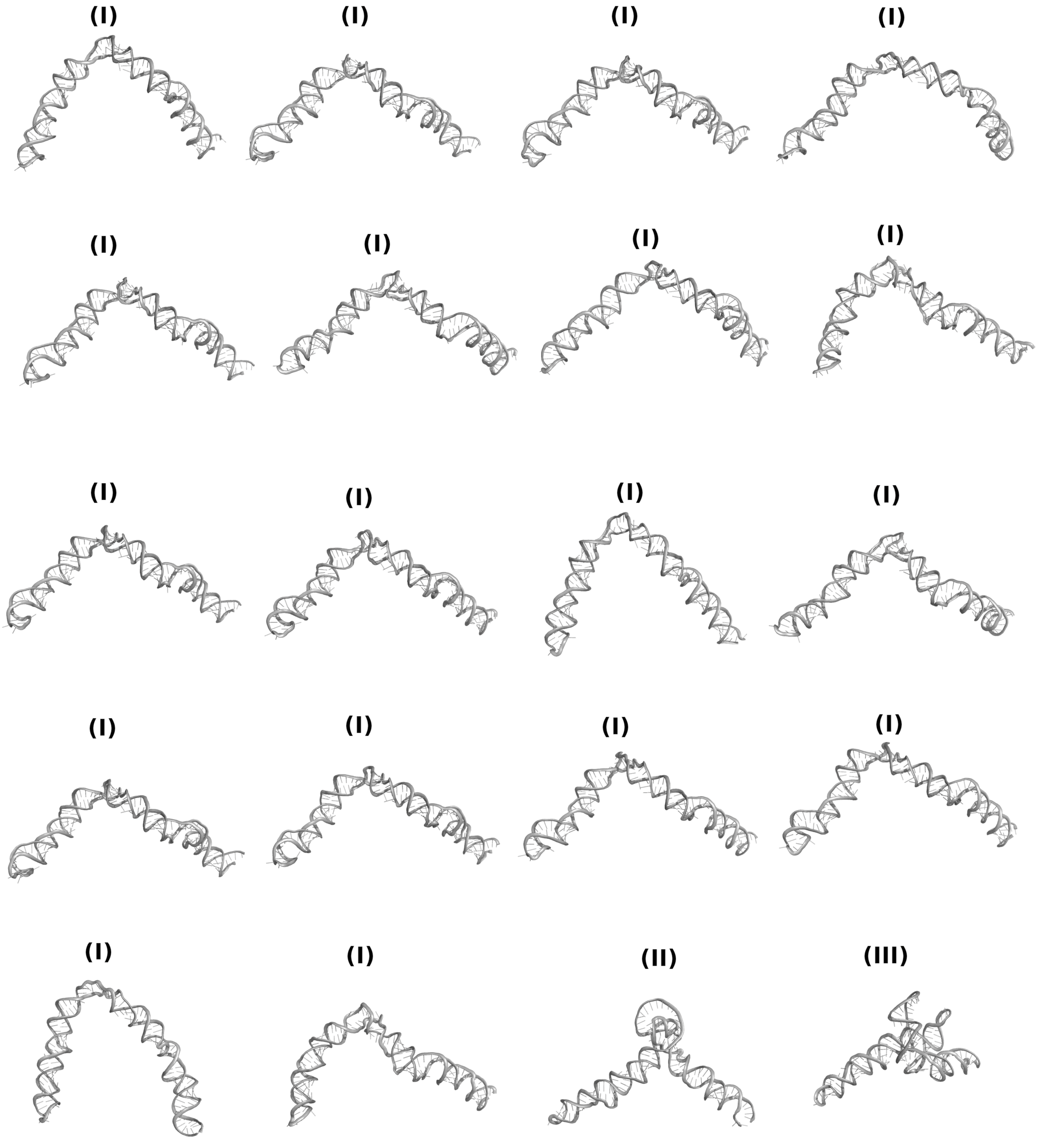

Supplementary Figure S3. A representative ensemble of free 6S models compatible with the corresponding SAXS data. 18/20 models correspond to Fold **I**, 1/20 to Fold **II** and 1/20 to Fold **III**.

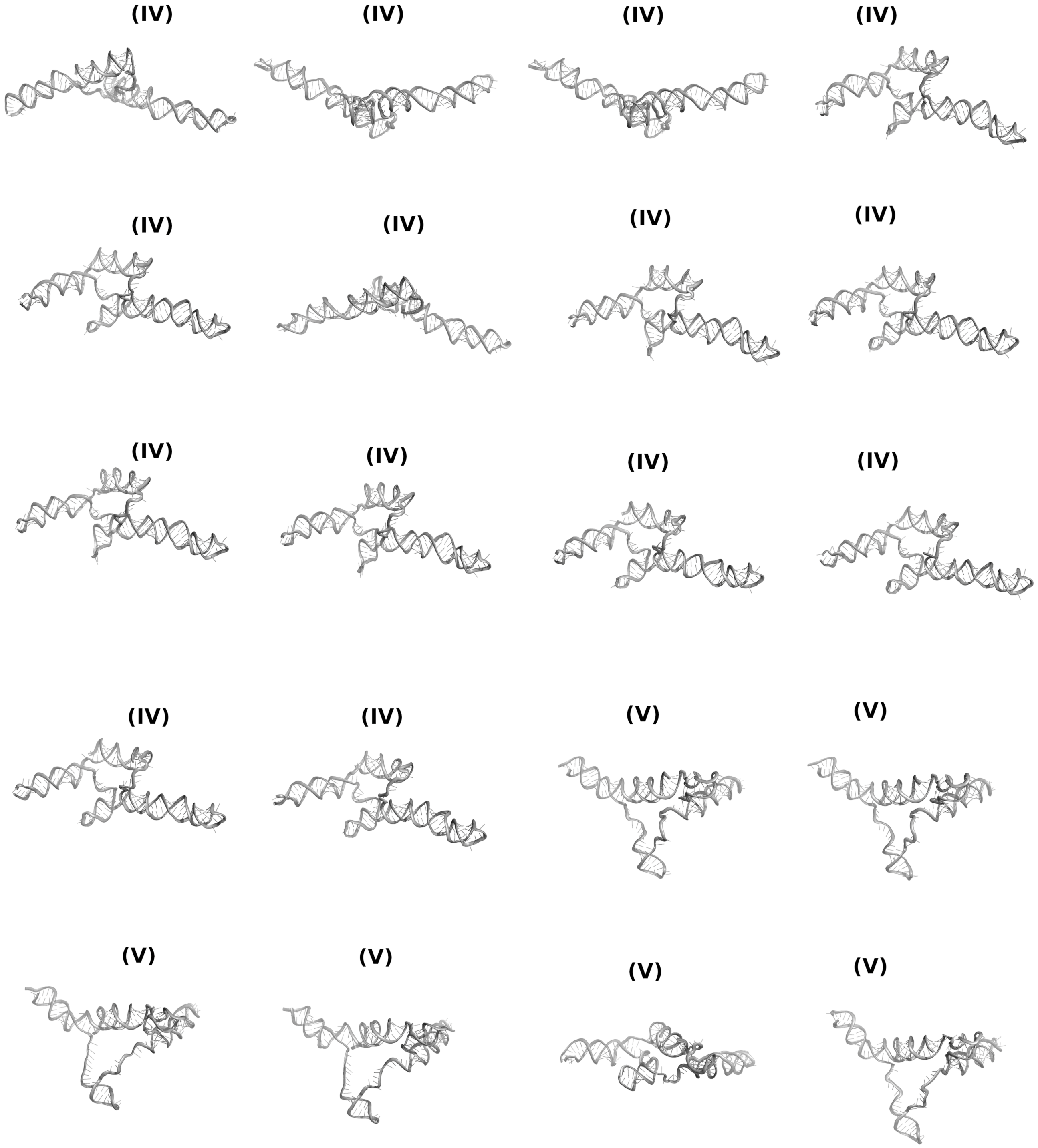

Supplementary Figure S4. A representative ensemble of 6S:pRNA models compatible with the corresponding SAXS data. 14/20 models correspond to Fold **IV** and 6/20 to Fold **V**.
